# Tumor Touch Imprints as Source for Whole Genome Analysis of Neuroblastoma Tumors

**DOI:** 10.1371/journal.pone.0161369

**Published:** 2016-08-25

**Authors:** Clemens Brunner, Bettina Brunner-Herglotz, Andrea Ziegler, Christian Frech, Gabriele Amann, Ruth Ladenstein, Inge M. Ambros, Peter F. Ambros

**Affiliations:** 1 Department of Tumor Biology, CCRI, Children’s Cancer Research Institute, St. Anna Kinderkrebsforschung, Vienna, Austria; 2 Institute of Clinical Pathology, Medical University Vienna, Vienna, Austria; 3 S2IRP, CCRI, Children’s Cancer Research Institute, St. Anna Kinderkrebsforschung, Vienna, Austria; 4 Department of Pediatrics, Medical University Vienna, Vienna, Austria; University of Pittsburgh Cancer Institute, UNITED STATES

## Abstract

**Introduction:**

Tumor touch imprints (TTIs) are routinely used for the molecular diagnosis of neuroblastomas by interphase fluorescence in-situ hybridization (I-FISH). However, in order to facilitate a comprehensive, up-to-date molecular diagnosis of neuroblastomas and to identify new markers to refine risk and therapy stratification methods, whole genome approaches are needed. We examined the applicability of an ultra-high density SNP array platform that identifies copy number changes of varying sizes down to a few exons for the detection of genomic changes in tumor DNA extracted from TTIs.

**Material and Methods:**

DNAs were extracted from TTIs of 46 neuroblastoma and 4 other pediatric tumors. The DNAs were analyzed on the Cytoscan HD SNP array platform to evaluate numerical and structural genomic aberrations. The quality of the data obtained from TTIs was compared to that from randomly chosen fresh or fresh frozen solid tumors (n = 212) and I-FISH validation was performed.

**Results:**

SNP array profiles were obtained from 48 (out of 50) TTI DNAs of which 47 showed genomic aberrations. The high marker density allowed for single gene analysis, e.g. loss of nine exons in the *ATRX* gene and the visualization of chromothripsis. Data quality was comparable to fresh or fresh frozen tumor SNP profiles. SNP array results were confirmed by I-FISH.

**Conclusion:**

TTIs are an excellent source for SNP array processing with the advantage of simple handling, distribution and storage of tumor tissue on glass slides. The minimal amount of tumor tissue needed to analyze whole genomes makes TTIs an economic surrogate source in the molecular diagnostic work up of tumor samples.

## Introduction

Tumor touch imprints (TTIs) are widely used for cytopathological examinations of tumor cells [1,2]. In addition, TTIs present an excellent material source for interphase fluorescence in situ hybridization (I-FISH) studies [3]. Besides the visualization of genomic aberrations by FISH techniques, DNA sequencing techniques to study single gene alterations can also be successfully performed on TTI slides [4–7]. A simultaneous analysis of 50 genes, for instance, was conducted by targeted sequencing [8]. Killian et al. were among the few authors to visualize whole tumor genomes by applying different array comparative hybridization platforms on archived Fine Needle Aspiration Cytology specimens as a source comparable to TTIs [9]. The authors described a number of structural aberrations with a resolution limited by the respective platform [9]. As TTIs can be stored for many years without markedly influencing the quality of extractable DNA [10], these slides are a promising source for molecular studies.

### Neuroblastoma (NB) molecular diagnosis

The International Neuroblastoma Risk Group has introduced a system for NB pretreatment risk classification and treatment stratification based on distinct biological and clinical features. These include stage, age, histology (i.e. grade of tumor maturation), *MYCN* gene copy number, status of the long arm of chromosome 11 and DNA ploidy level of the tumor cells [11]. Thus, the classification is in large parts based on the known association of certain genetic aberrations with aggressive tumor growth and an unfavorable clinical outcome [11]. The current European Low and Intermediate risk study (LINES) even goes a step further by also using the information on the presence or absence of 7 segmental chromosomal aberrations (SCAs > 3Mb) in addition to the *MYCN* status for therapy stratification [12]. Thus, information on *MYCN* copy number and certain SCAs, i.e. deletions at 1p, 3p, 4p and 11q and/or gains at 1q, 2p or 17q, are relevant for decision making when assigning a patient to the appropriate therapy regimen [13]. Besides these relatively large genomic aberrations, deletions aberrations of single genes or parts thereof or point mutations, e.g. *TERT* [14], *ALK* [15–17], *ATRX* [18,19] and *ARID1A* [20], have also recently been described for NB. The complexity of the aberrations present at different genomic loci and the possible combinations thereof demand a detailed investigation of the whole genome. A first-line *MYCN* copy number evaluation by I-FISH combined with an ultra-high density SNP (UHD SNP) array analysis fulfills the current need to categorize NB tumors into defined genomic subtypes [21].

### UHD SNP array platform

UHD SNP arrays are an oligonucleotide hybridization microarray platform that can be used to detect copy number variations as well as allelic imbalances simultaneously. The Affymetrix Cytoscan HD platform comprises approximately 2 million copy number and 750 thousand SNP markers across the whole genome. In addition to large aberrations on the chromosomal level, the ultra-high density of more or less randomly arranged markers allows for the analysis of single genes or parts thereof.

### Aim

In this feasibility study, we examined the suitability of TTIs as a source for the identification of amplified sequences, SCAs, as well as minor genomic aberrations that affect single genes or parts thereof using a UHD SNP array platform. We focused on NB samples since the LINES study is one of the very first clinical trials worldwide to explore the usability of a series of SCAs in addition to information on *MYCN* copy number in the decision making process [12] and the feasibility of identification of possible new prognostic genomic changes. Furthermore, we tested whether TTIs from other tumor entities can also be used to reliably identify genomic changes.

## Material and Methods

### Tumor touch imprints

TTIs were either received from other laboratories or prepared from fresh or fresh frozen tumor pieces by touching them very gently against a glass slide held with a sterile forceps. This procedure had to be performed carefully in order to avoid squeezing since shear forces may disrupt cells. The slides were placed in airtight plastic containers and were stored at -20°C. Before usage, slides were thawed at room temperature while the storage containers were kept closed to avoid water condensation on the glass slides.

The number of glass slides used for DNA extraction varied from 1 to 8 slides per tumor, depending on the availability of slides and visual examination of the cell density, which varied greatly between slides ranging from scattered cell aggregates to macroscopically visible tumor cell clumps.

### Source material

For the quantification of the cell number per slide, two TTIs were stained with 4’,6-diamidino-2-phenylindole (DAPI; Sigma-Aldrich Co.). Cell nuclei were counted with an automatic fluorescence microscope (Zeiss Axioplan 2 imaging) with a digital camera (MetaSystems). Image analysis was done using the Metafer 4 software module RCDetect (MetaSystems, version 3.6.7). To calculate the expected amount of DNA per slide, we multiplied the mass of a diploid human genome (approx. 6 pg) with the number of nuclei per slide.

### DNA extraction from tumor touch imprints

All together, we extracted DNA from 50 tumor samples from 44 patients. The cohort comprised 40 patients diagnosed with NB. For three patients (P06, P16 and P26), DNA was extracted from multiple tumor samples. The non-NB tumors from four patients were of different diagnoses and included: one desmoplastic small-round-cell tumor, one Ewing tumor, one medulloblastoma, and one osteosarcoma. In cases with a low cell number per slide, cells from different slides from the same tumor were pooled prior to DNA extraction.

A sterile polypropylene swab was drenched in Phosphate Buffered Saline (PBS) and the slides were wiped with it. The swab tip was cut from the handle and placed into a 1.5 mL microcentrifuge tube, 1 mL of PBS added and the tube vortexed for 10 s. The shortened swab (the swab was shortened sufficiently to allow closing the tube before centrifugation) was transferred inversely to a fresh 1.5 mL tube and centrifuged for 1 min at 16,100 rcf. The cell solution drain was pooled with the initial PBS washing solution. This centrifugation step was repeated once. The pooled cells were centrifuged at 16,100 rcf for 3 min. DNA was extracted from the resulting cell pellet following an adapted high salt protocol by Miller [22], i.e. 300 μL Lysis Buffer (10 mM Tris-HCl pH 8.0, 400 mM NaCl, 2 mM EDTA), 3 μL proteinase K (20 mgmL^-1^, Carl Roth, Karlsruhe, DE-BW) and 13 μL SDS (20% solution) were added and incubated at 56°C overnight. The proteins were precipitated with 100 μL of 6 M NaCl solution and centrifuged for 30 min at 3,500 rcf. The supernatant was transferred into a fresh 1.5 mL tube and centrifuged for 3 min at 16,100 rcf. The new supernatant was again transferred into a fresh 1.5 mL tube and the DNA was precipitated with 1 mL absolute ethanol and centrifuged for 15 min at 16,100 rcf. After removing the supernatant, the DNA pellet was air dried by opening the tubes for 30 min at RT. The DNA was dissolved in a minimum of 10 μL TE buffer (pH 8.0, Applichem GmbH, Darmstadt, DE-HE) overnight. Double stranded DNA (dsDNA) was quantified with the Qubit dsDNA HS Kit (Thermo Fisher Scientific, Waltham, US-MA).

### SNP array analysis

The Cytoscan HD SNP array platform (Affymetrix, Santa Clara, US-CA) was used for the generation of tumor profiles according to the assay’s protocol. The 50 extracted DNAs were diluted to a working concentration of 50 ng μL^-1^ based on Nanodrop concentration values. Solutions with concentrations below 50 ng μL^-1^ were used undiluted. Quality control gels were run at 120 V for 50 min and stained with 3X GelRed (Biotium, Hayward, US-CA). Cytoscan data were analyzed with the Chromosome Analysis Suite software (Affymetrix, ChAS; version 2.1). The annotation version NA33 used by the ChAS software is based on the February 2009 human reference sequence GRCh37 (hg19) [23]. The UHD SNP array data were interpreted as described previously [24]. Circos Plots were generated with the Circos Software [25].

### Assessment of array data quality

SNP array data quality was assessed with the internal array quality control parameter “Median of the Absolute Values of all Pairwise Differences” (MAPD), which estimates the variability of log2 ratios over the complete array with robustness against high biological variability as frequently found in tumor DNA samples. MAPD values below 0.25 were considered to be indicative for good quality [26].

### Data quality comparison between TTIs and fresh or fresh frozen tumors

The MAPD mean value of the TTI samples was compared to the MAPD mean value of an independent cohort of 212 randomly chosen fresh or fresh frozen NB tumors from which DNAs were extracted and processed according to the same DNA extraction method and Cytoscan HD protocol as for TTIs.

### Statistics

To test for linear dependencies, *t-*tests based on Pearson’s product moment correlation coefficient (*r*) were used. For mean comparison, we used Welch’s *t*-test. *P* values < 0.05 were considered to be significant. Statistics were calculated using the software *RStudio* (version 0.97.551) [27] that is based on the statistical computing language R [28].

### Data validation by I-FISH

To validate TTI SNP array data, we compared our findings with I-FISH data from corresponding tumor samples. I-FISH experiments on appropriate TTIs were done as already described [29]. The used I-FISH probes are listed in S1 Table.

### Ethics

Ethical permission for the diagnostic analysis was granted by the local ethics committee of the Medical University Vienna (Ek-Nr: 920/2011). The participants provided a written informed consent.

## Results

### TTI DNA extraction

The concentrations of the DNA solutions measured with Nanodrop ranged from 0.97 ng μL^-1^ to 3.94 μg μL^-1^ with a mean of 620.3 ng μL^-1^ and a median of 155.1 ng μL^-1^ (n = 50). The concentrations of dsDNA measured with the Qubit method ranged from 0.92 ng μL^-1^ to 4.18 μg μL^-1^ with a mean of 603.4 ng μL^-1^ and a median of 97.2 ng μL^-1^ (n = 26). Although not all DNA concentrations met the recommended Cytoscan minimal assay working concentration of 50 ng μL^-1^ (5 μL starting volume; 250 ng total DNA), DNAs from all 50 TTIs were processed.

### Cell counting

The fluorescence based quantification of DAPI positive nuclei revealed 79,967 nuclei for NB08 and 1,917 nuclei for NB39. In theory, this number of nuclei counted in NB08 and NB39 would result in 523.78ng and 12.35ng DNA, respectively. After DNA extraction 244 ng and 11.1 ng, respectively, of total DNA were obtained from the two TTIs.

### SNP array data

SNP array profiles were obtained from 44 NB (38 NB patients) and from 4 other pediatric tumors. DNAs of two NB patients failed PCR amplification and thus SNP profiles were not generated. One NB sample showed a so-called flat profile lacking any type of larger chromosomal aberration. In all other samples either amplifications, numeric chromosome aberrations (NCAs), SCAs or a combination of aberrations were found (47 out of 48) in varying constellations. Additionally, copy neutral losses of heterozygosity (cnLOHs), including uniparental disomies (UPDs) or uniparental trisomies (UPTs), affecting whole chromosomes were observed. Aberrant copy numbers of single genes were detected as well. A detailed list of all aberrations can be found in S2 Table (NB) and S3 Table (non-NB).

### Influence of long term storage on array data quality

**Fig 1:**
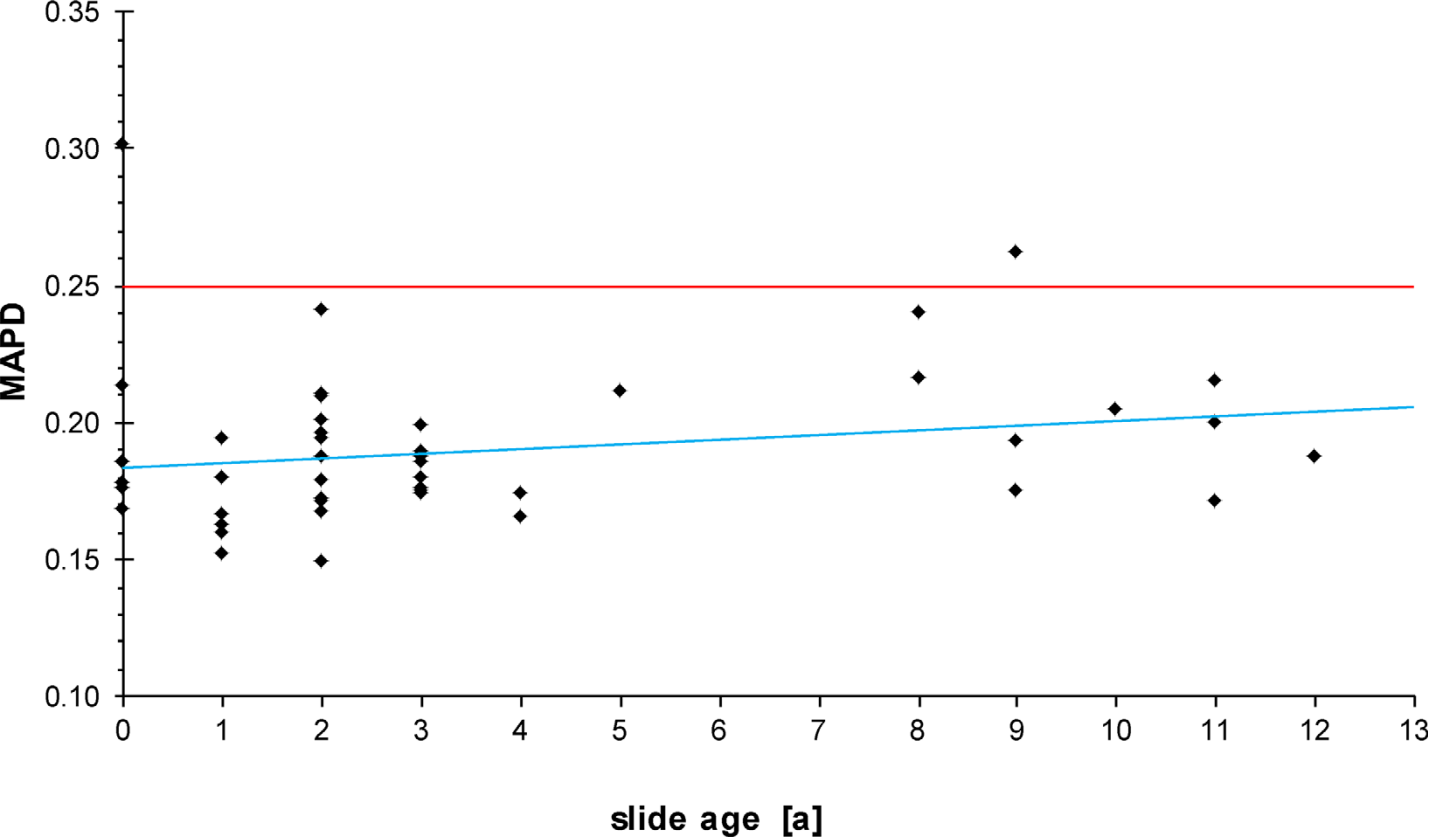
Influence of slide age on array data quality (MAPD values). The linear trend line is shown in blue. MAPD values below 0.25 (red line) are considered to represent a good quality.

To analyze if the age of the TTI slide had an influence on the array data quality, we correlated the different storage times with the MAPD values. The data indicated that the array data quality was independent of the slide age (*t* = 1.457, *P* = 0.152, r = 0.21) as shown in Fig 1.

### Data quality comparison between TTIs and fresh or fresh frozen tumors

MAPD values of the TTI SNP arrays ranged from 0.149 to 0.315 with a median of 0.187 and a mean of 0.195. MAPD values from the fresh or fresh frozen cohort ranged from 0.147 to 0.469 with a median of 0.176 and a mean of 0.188. The mean MAPD values showed no significant difference (*t* = 1.313, *P* = 0.193).

### NB SNP array profiles

**Fig 2:**
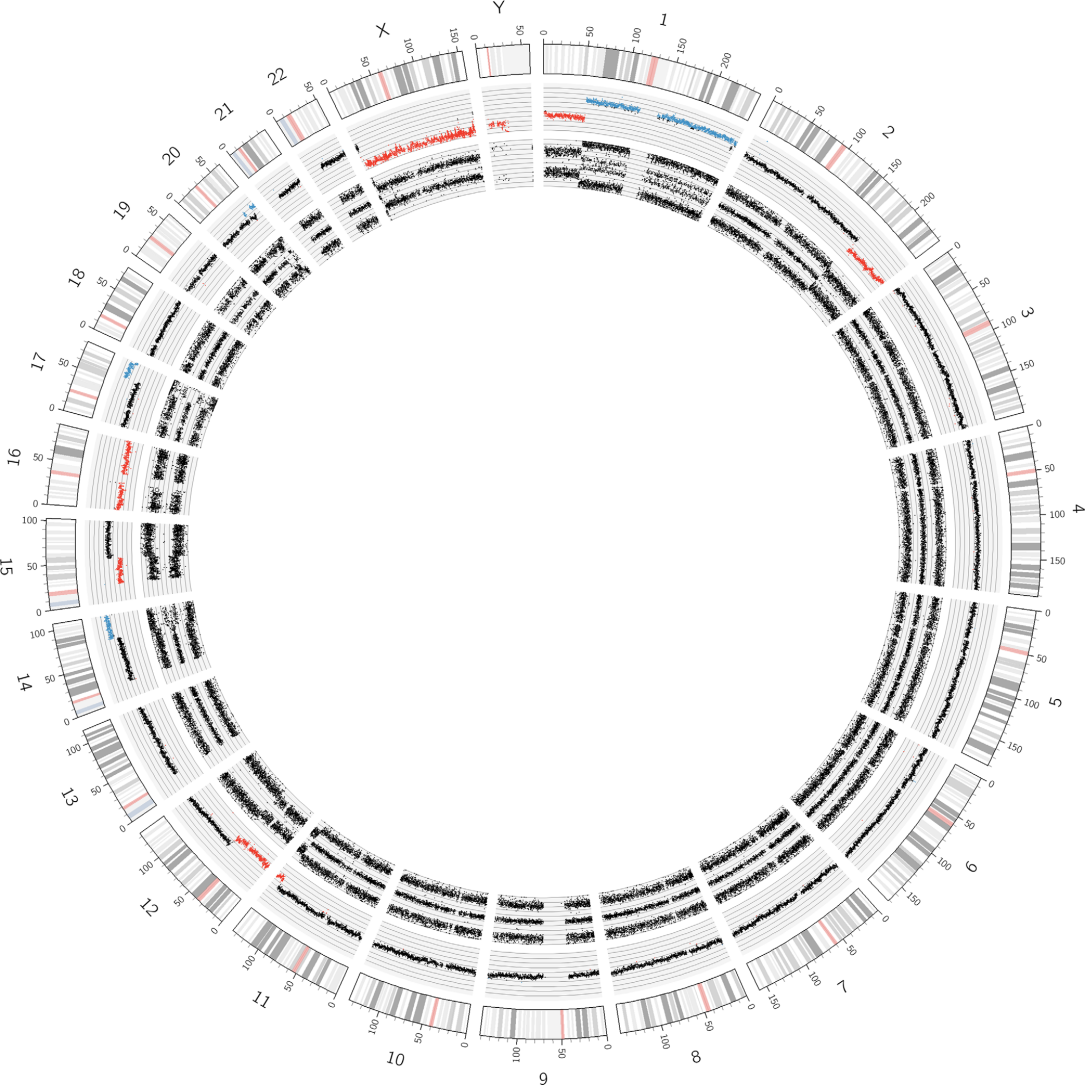
Circos Plot of copy number and SNP probe data for sample NB05. The tracks are from outside inwards: chromosome numbers, chromosomal positions in Mb, copy number and allele patterns. Copy number gains and losses are shown in blue and red, respectively. SCAs resulting in deletion of the concerned region where seen at 1p, 2q, 11q, 12p/q and 15q. Gains were found at: 14q, 17q and 20q. Chromosomes 12 and 15 show partial UPDs in addition to the before mentioned deletions. Chromosome 16 discloses a monosomy with a typical two band allele pattern (allele configurations A0 and B0). Most autosomes (3, 4, 5, 6, 7, 8, 9, 10, 13, 18, 19, 21 and 22) show a continuous disomic copy number profile with a normal three band allele pattern (allele configurations AA, AB and BB). A *MYCN* amplification is present but hardly visible at the short arm of chromosome 2 (small blue dot). The sex chromosomes show monosomies (except for the pseudoautosomal regions on the X chromosome) indicating that this profile belongs to a male patient.

To verify whether evaluable and well-interpretable UHD SNP arrays are suitable for the genetic diagnosis of NBs, we analyzed the copy number status and allele specific probe information of whole chromosomes, chromosomal segments, and single genes. The SNP array profile of sample NB05 is shown exemplarily in Fig 2.

**Fig 3:**
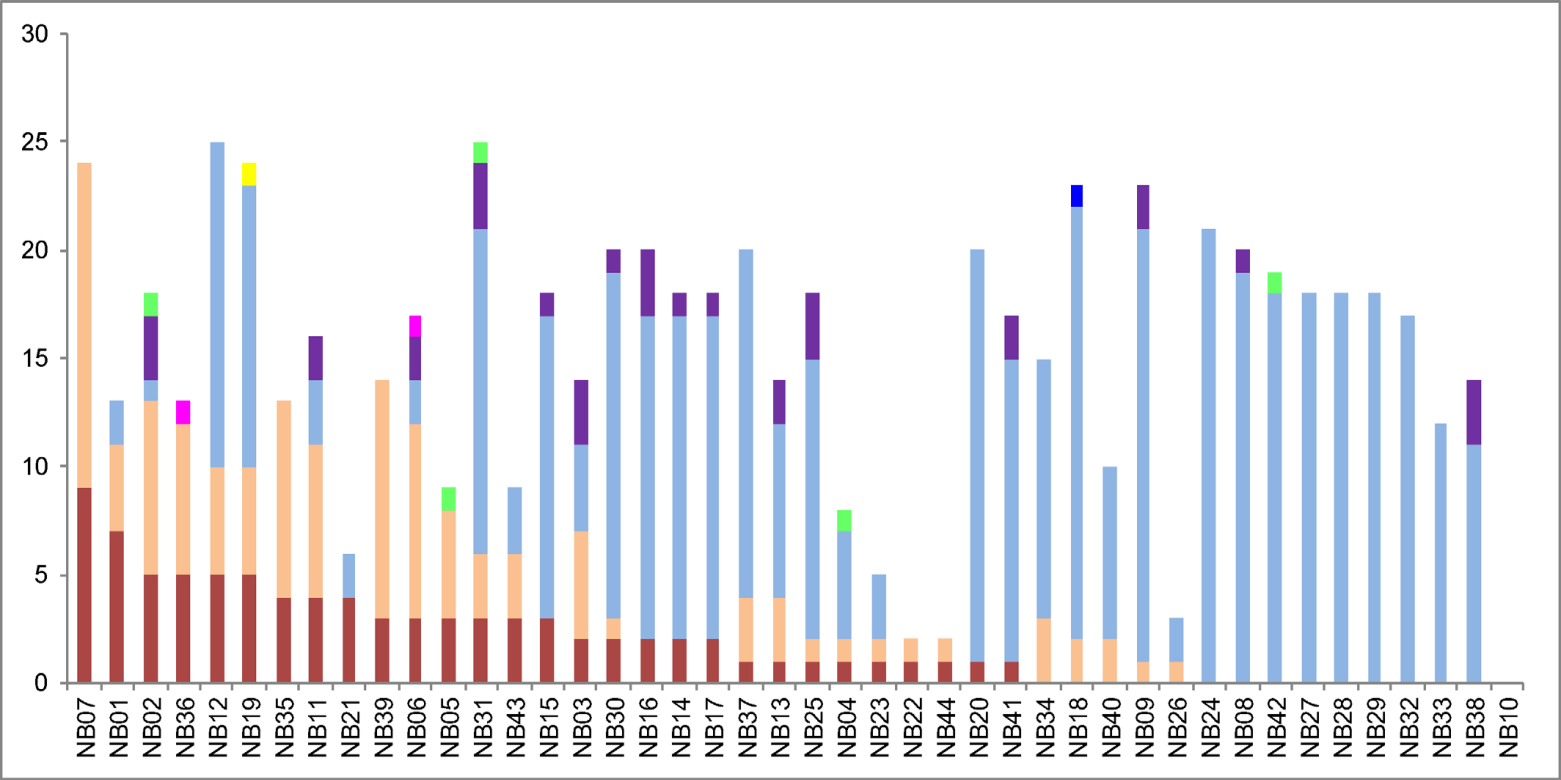
Number and type of chromosomal aberrations found in the cohort of 44 NB TTIs. Each bar represents one tumor. The number of NB typical segmental chromosomal aberrations (SCAs) is shown in red. SCAs with so far unknown prognostic relevance are shown in orange and numerical chromosomal aberrations (NCAs) are shown in blue. Whole chromosome copy neutral losses of heterozygosity are shown in purple. Presence of *MYCN* gene amplifications is shown in green, intragenic *ATRX* gene deletions in pink, hyper-rearrangements in dark blue and chromothripsis in yellow.

To visualize the different genomic aberrations found in NB, we attributed the most frequently occurring aberrations to a certain color (Fig 3) and ranked them from left to right according to the presence and frequency of aberrations in the following order: i) NB-typical SCAs in red ii) NB-atypical SCAs in orange iii) NCAs in blue iv) whole chromosome cnLOHs in purple. *MYCN* amplification (MNA) is indicated in green, an intragenic *ATRX* deletion in pink, a hyper-rearrangement in dark blue and chromothripsis in yellow color. cnLOHs affecting whole chromosomes were found exclusively in tumors with gains or losses of whole chromosomes, i.e. with a hyperdiploid DNA content. MNA was associated with all genomic subtypes, except for case NB42, where MNA was the only structural aberration. All other *MYCN*-amplified tumors showed co-occurrence with NCAs and/or SCAs. In seven tumors, only structural aberrations without whole chromosome gains were found. In addition to copy number aberrations, the SNP array provides information on the allele status. In 17 profiles whole chromosome cnLOHs (UPDs or UPTs) were present (Fig 3).

**Fig 4:**
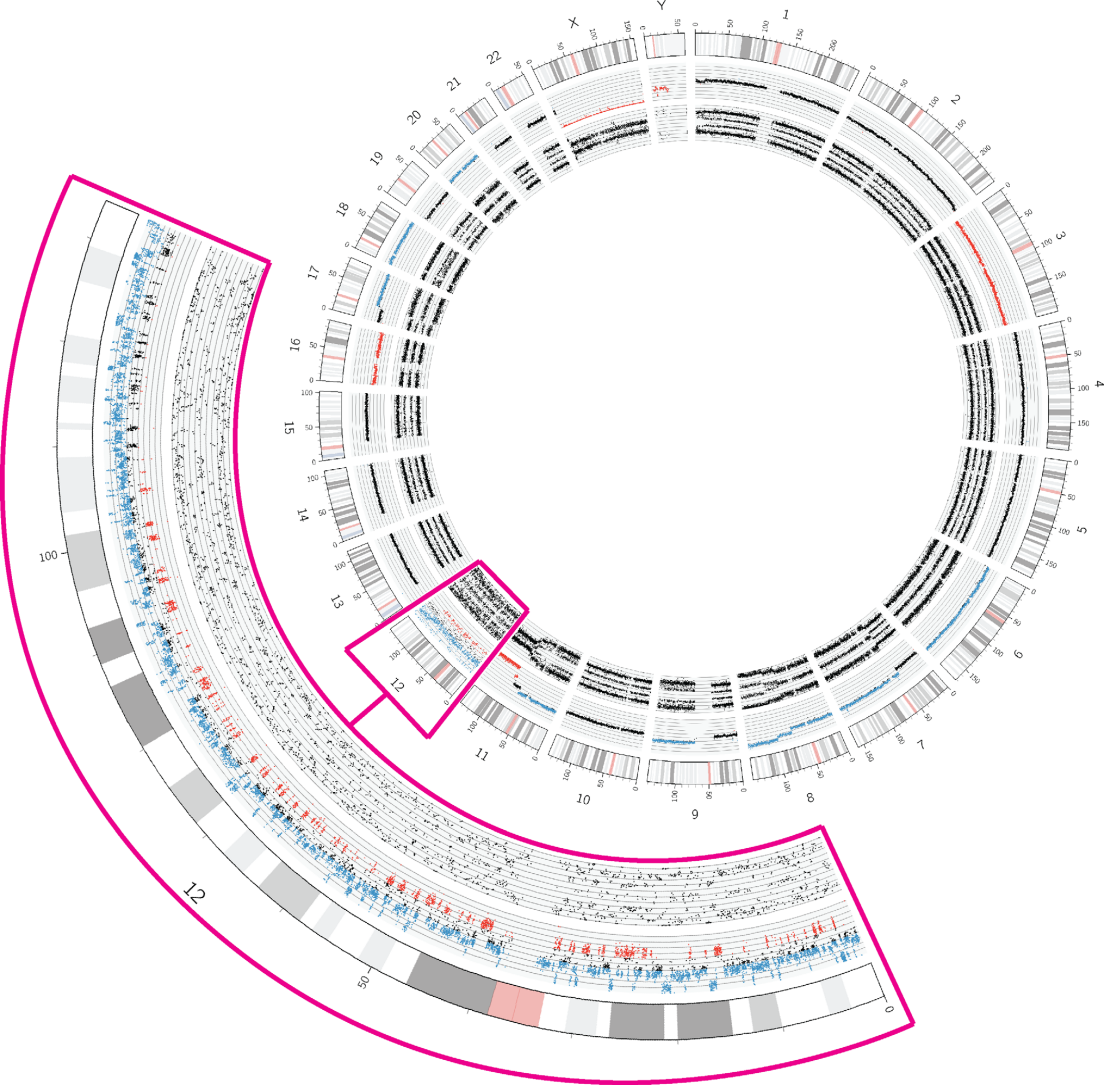
Circos plot of sample NB19 demonstrating chromothripsis. The tracks are from outside inwards: chromosome numbers, chromosomal positions in Mb, copy number and allele patterns, respectively. Copy number gains and losses are colored blue and red, respectively. The enlarged segment marked in pink shows a highly rearranged chromosome 12 with more than 200 breakpoints indicating chromothripsis. Additionally, a gain of the distal part of the q-arm of chromosome 12 is present. SCAs were identified on chromosomes 1, 2, (both SCAs only in a subpopulation of cells) 7, 8, 9, 11 (three SCAs) and 17.

The tumor genome of sample NB19 showed a highly rearranged chromosome 12 with more than 200 breakpoints fitting the criteria for chromothripsis (Fig 4). Other structural aberrations affected chromosomes 1, 2, 7, 8, 9, 11 and 17. MNA was not detected. The histological report from this tumor indicated a stroma poor neuroblastoma. The histological examination of the second look surgery 10 months later revealed a nodular ganglioneuroblastoma with morphological different regions showing signs of tumor maturation and many regions with undifferentiated neuroblastoma tissue.

### Single genes

**Fig 5:**
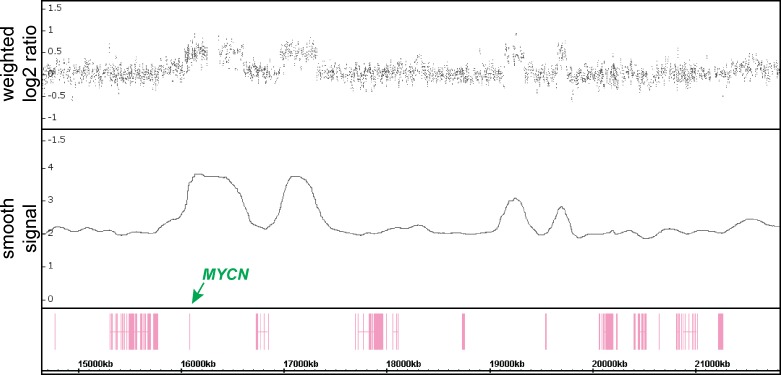
2p copy number analysis of NB02. This SNP array profile shows a part of chromosome 2 (approx. 15–22 Mb). In the upper track the weighted log2 ratios, in the middle track the smoothed copy number signals and in the lower track the spatial position of annotated genes along the chromosome are shown. The smoothed copy number profile shows four peaks (representing the amplicon) with an average smoothed signal of approx. 3.5 copies including the *MYCN* gene. The co-amplified regions contain no annotated genes.

In four cases (9.1%), the *MYCN* gene specific probes and adjacent copy number loci exceeded the generally accepted (≥ times in relation to the chromosome 2 copy number) threshold for *MYCN* gene amplification. Profiles NB04, NB05, NB31 and NB42 showed copy number values of 41, 35, 30 and 21, respectively. Profile NB02 showed a gain of the *MYCN*-specific and adjacent probes with a mean value of approximately 3.5 copies (Fig 5). Our assumption that this tumor shows an intratumoral heterogeneous MNA (hetMNA) was confirmed by I-FISH (data not shown). Thus, in a technique relying on extracted bulk DNA such as SNP array, the high number of *MYCN* copies in single cells is equaled out by a high number of non-amplified tumor cells, resulting in a copy number status below the *MYCN* amplification threshold.

**Fig 6:**
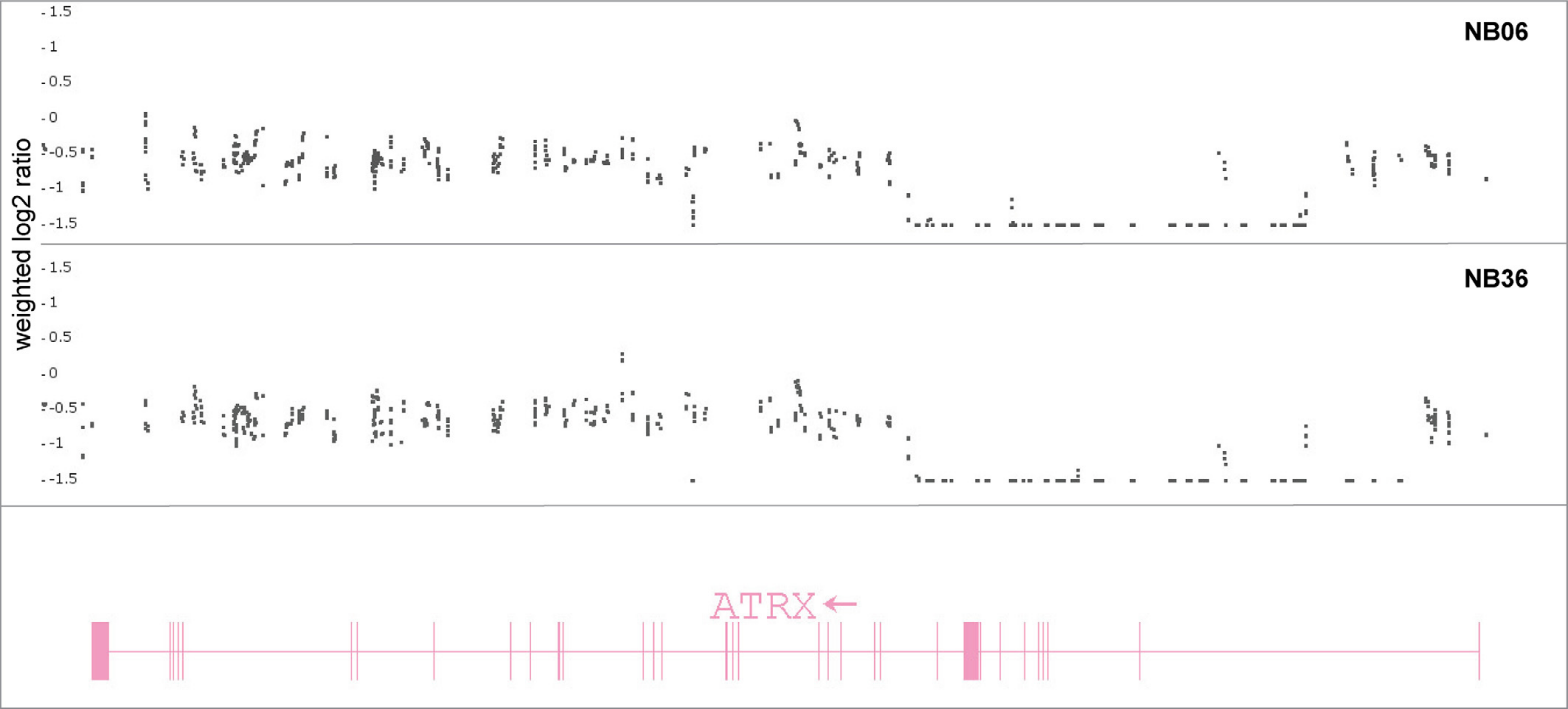
Partial *ATRX* nullisomy in NB samples NB06 and NB36. Exons 2–10 of the *ATRX* gene (transcript variant 1; NCBI RefSeq accession number NM_000489) are deleted in both NB samples (NB06 upper panel, NB36 middle panel). The proximal breakpoints are located at the same chromosomal position in both samples (76,925 kb), however, the lengths of the deletion differed in both samples (~ 80 kb for NB06 and ~ 100 kb for NB36). The lower track shows the spatial assembly of the *ATRX* gene, the exons are indicated by vertical lines or blocks.

In addition to amplification of the *MYCN* gene, other genes were found to be structurally altered. A partial *ATRX* deletion was detected in two cases (NB06 and NB36) with an identical proximal breakpoint at chromosomal position 76,925 kb. The aberration lengths differed between approximately 80 kb for NB06 and approximately 100 kb for NB36. However, in both cases the exons 2–10 of transcript variant 1 were deleted as shown in Fig 6.

### SNP array data from other tumor entities

In addition to NB samples, TTIs from one Ewing tumor (ES01), one desmoplastic small round cell tumor (DSRCT01), one osteosarcoma (OS01) and one medulloblastoma (MB01) were analyzed. As listed in S3 Table, we identified within tumor MB01 NCAs of chromosomes 8, 14, 19 and 22 and a cnLOH (UPT) of the whole chromosome 4. The tumor cells isolated from TTIs from DSRCT01 showed SCAs on chromosomes 1 and 21 and NCAs on chromosomes 2, 3 and 8. ES01 showed no NCAs but SCAs on chromosomes 1 and 10 as well as a hyper-rearranged region (approx. 15 Mb) at the proximal part of the long arm of chromosome 22. This region included an interstitial stretch of CNLOH (UPD; approx. 3 Mb) that included the complete *EWSR1* gene. Another interstitial stretch (approx. 900 kb) within the 22q hyper-rearrangement was completely deleted (nullisomy) and included the complete *SMARCB1* gene. OS01 showed no NCAs but structural genomic aberrations affecting chromosomes 3, 6, 7, 8, 12, 13, 15, 16, 17, 19, 20 and 21. Hyper-rearrangements where found on chromosomes 3, 4, 12, 17 and 19. In addition, two amplicons were detected. One was located within the hyper-rearranged region on chromosome 17 and included the complete *NCOR1* gene (approx. copy number 21). The other amplicon at chromosomal position 19p13.2 included the complete *INSR* gene (approx. copy number 41).

### SNP data validation by I-FISH

Corresponding I-FISH data were available for 37 TTI samples. All aberrations detected by I-FISH (i.e. MNA, loss at 1p36 and gain of 17q) were also seen in the SNP array profiles. In addition, since the exact DNA content of the tumor was frequently unknown, the information obtained by I-FISH on the copy number status of chromosomes 1, 2 and/or 17 allowed for a better interpretation of the copy number changes seen in the SNP array data. Thus, the correct chromosome somy levels for these chromosomes could be determined since the ChAS software algorithm sets the disomic baseline of aneuploid tumors not correctly.

The I-FISH results from the TTIs from ES01 and DSRCT01 indicated a rearrangement of the *EWSR1* gene in both tumors. However, as reciprocal translocations are only detectable by SNP array when they are accompanied by gains or losses of chromosomal material at or close to the breakpoint, the results obtained with the two techniques do not necessarily match each other. In ES01, the *EWSR1* gene was altered by an UPD event but an intragenic alteration affecting this gene was not detectable. In DSRCT01, the *EWSR1* copy number status appeared normal in the SNP profile without any hint of alteration. Detailed I-FISH results are listed in S2 Table and S3 Table.

## Discussion

This study successfully demonstrates the usability of TTIs as an excellent source for whole genome analysis by applying the UHD SNP array technique. Thus, TTIs can be considered as a valuable surrogate source for tumor tissue. Especially during the histopathological preparation, the production of TTIs does not add another laborious procedure besides the snap freezing of tumor tissue, which should be done in any case. The amount of cells on TTIs varied greatly between samples, resulting in a high variation of extracted DNA quantities per slide, as already reported in other studies [6] [7] [8] [10]. Compared to fresh or fresh frozen solid tumor pieces, the cell number on TTIs can be low, which can result in a limited DNA quantity. According to the Qubit data, the lowest concentration of dsDNA that could be processed successfully was 0.92 ng μL^-1^ (sample NB10; 4.59 ng dsDNA per slide). However, the data quality measure, i.e. the MAPD value, of sample NB10 was still below the 0.25 threshold, indicating a good quality. All in all, the MAPD values show that the quality of TTI SNP profiles was comparable to SNP profiles from fresh or fresh frozen tumor samples. Thus, storage of the slides for up to 12 years did not significantly influence the SNP array data quality. This eliminates the need for technically challenging liquid nitrogen storage systems. The DNA of two samples (NB46 and NB47) could not be amplified by PCR. However, the reason for this failure remains unclear as quality and quantity of the DNA samples were comparable to samples for which the SNP array procedure worked satisfactorily.

The data indicate that the UHD SNP array platform is a reliable tool for the analysis of TTIs. First results indicate that whole genome sequencing/low coverage also produces clear copy number results. This is of special interest for minimal invasive procedures, e.g. core needle (tru-cut) or fine needle aspiration biopsy, where only small tissue samples are obtained. Taking more biopsies from geographically distinct regions in individual tumors may help to reduce the risk of overlooking genetically different areas in heterogeneous tumors. In addition to the easiness of producing TTIs, they have the advantage of uncomplicated shipment, as they do not require any frozen/cooled shipment solutions. This facilitates the sending of tumor material to reference laboratories and the sharing of tumor material for collaborative studies.

The wealth of detailed information on the genomic changes provided by the UHD SNP array platform from TTI samples was remarkable. Aberrations affecting whole chromosomes (NCAs) or parts thereof (SCAs) could be detected as well as aberrations affecting single genes. In case of the *ATRX* gene, even deletions concerning certain exons were identified in two cases. However, it has to be noted that such an in-depth analysis depends on the marker density within the respective gene of interest, and not all genes are covered as densely as in the case of the *ATRX* gene. The visualization of other aberrations—such as the hyper-rearranged region found on chromosome 12 in sample NB19 indicating chromothripsis—can also be facilitated by the dense marker distribution of the applied array.

For the genetic workup of NB tumors, the whole genome approach by UHD SNP array worked extremely well. All aberrations currently relevant for the molecular prognostication of NB, e.g. *MYCN* amplifications, SCAs, NCAs and *ATRX* deletions, were detected by this platform and aberrations with unknown prognostic significance were also identified beyond doubt. In one NB sample, the rather rarely occurring intra-tumor heterogeneity of MNA was found. The identification of heterogeneous MNA [30] [31] may be crucial for an adapted therapy stratification and an adequate follow-up care. However, the unambiguous identification of heterogeneous MNA by SNP array analysis depends on the number of *MYCN*-amplified cells contained in the appropriate sample, i.e. on TTIs, and false negativity may occur due to the detection limit of techniques based on extracted bulk DNA. Thus, the analysis of distinct tumor cell clones without the application of I-FISH or enrichment techniques, e.g. tumor cell enrichment by magnetic beads [32] or extraction of defined regions in immune-cytologically stained slides, could be challenging. As expected, we did not find any discordance between the data concerning *MYCN* copy number, integrity of 1p36 or 17q gain obtained by I-FISH and the copy number data from the SNP array of corresponding samples.

As SNP array data obtained from TTIs allow detailed and reliable insights into the genomic landscape of NB and other tumor entities, TTIs can be considered a reliable tool to unravel the genomic details of solid tumors and can aid in the therapy stratification process as, e.g., is currently done in the clinical LINES (European Low and Intermediate Risk, ClinicalTrials.gov Identifier: NCT01728155) study protocol where the risk group assignment is based on clinical, histological and genomic information.

Importantly, the use of TTIs for pan-genomic analyses is not restricted to NB samples since we were able to detect genetic aberrations in four other pediatric tumor entities as well. All in all, TTIs represent a reliable source for genomic analysis in the field of molecular cancer diagnosis and research applications.

## Supporting Information

S1 Table I-FISH probes. (XLSX)

S2 Table Detailed SNP array and I-FISH results of neuroblastoma samples. This table lists segmental chromosomal aberrations typical for neuroblastomas (SCAs) and SCAs of so far unknown diagnostic relevance (atypical SCA), numerical chromosomal aberrations (NCAs), and aberrant copy numbers of single genes of selected chromosomes. Corresponding I-FISH results are included in the far right column.
(XLSX)

S3 Table SNP array and I-FISH results of non-neuroblastoma TTI samples. This table presents SCAs, NCAs, wc cnLOHs and altered single genes of possible interest in one Ewing tumor (ES01), one desmoplastic small round cell tumor (DSRCT01), one medulloblastoma (MB01) and one osteosarcoma (OS01).
(XLSX)
